# Preliminary evaluation of a smartphone application (DelApp) for identification of delirium in sub-Saharan Africa

**DOI:** 10.1017/neu.2023.29

**Published:** 2023-06-22

**Authors:** Stella-Maria Paddick, Editruda Gamassa, Nuru Mwaluwinga, Grace Lewis, Ashanti Duinmaijer, Sarah Urasa, Laura Tucker, Elizabeta Blagoja Mukaetova-Ladinska, Glynis Cosker, Marieke Dekker, Aloyce Kisoli, Jane Cletus, Caroline Lissu, Catherine Dotchin, William K. Gray, Richard Walker

**Affiliations:** 1 Institute of Translational and Clinical Medicine, Newcastle University, Newcastle upon Tyne, UK; 2 Cumbria Northumberland Tyne and Wear NHS Foundation Trust, Newcastle upon Tyne, UK; 3 Kilimanjaro Christian Medical University College, Moshi, Tanzania; 4 Charité - Universitätsmedizin Berlin CVK: Campus Virchow-Klinikum Institute of Tropical Medicine and International Health, Berlin, Germany; 5 Haydom Lutheran Hospital, Mbulu, Manyara, Tanzania; 6 The London School of Hygiene & Tropical Medicine, London, UK; 7 University of Leicester, Leicester, UK; 8 Hai District Hospital, Boman’gombe, Kilimanjaro, Tanzania; 9 Institute of Population Sciences, Newcastle University, Newcstle upon Tyne, UK; 10 Northumbria Healthcare NHS Foundation Trust, North Shields, UK

**Keywords:** delirium, screening, Africa, cognitive assessment

## Abstract

**Objective:**

In sub-Saharan Africa, there are no validated screening tools for delirium in older adults, despite the known vulnerability of older people to delirium and the associated adverse outcomes. This study aimed to assess the effectiveness of a brief smartphone-based assessment of arousal and attention (DelApp) in the identification of delirium amongst older adults admitted to the medical department of a tertiary referral hospital in Northern Tanzania.

**Method:**

Consecutive admissions were screened using the DelApp during a larger study of delirium prevalence and risk factors. All participants subsequently underwent detailed clinical assessment for delirium by a research doctor. Delirium and dementia were identified against DSM-5 criteria by consensus.

**Results:**

Complete data for 66 individuals were collected of whom 15 (22.7%) had delirium, 24.5% had dementia without delirium, and 10.6% had delirium superimposed on dementia. Sensitivity and specificity of the DelApp for delirium were 0.87 and 0.62, respectively (AUROC 0.77) and 0.88 and 0.73 (AUROC 0.85) for major cognitive impairment (dementia and delirium combined). Lower DelApp score was associated with age, significant visual impairment (<6/60 acuity), illness severity, reduced arousal and DSM-5 delirium on univariable analysis, but on multivariable logistic regression only arousal remained significant.

**Conclusion:**

In this setting, the DelApp performed well in identifying delirium and major cognitive impairment but did not differentiate delirium and dementia. Performance is likely to have been affected by confounders including uncorrected visual impairment and reduced level of arousal without delirium. Negative predictive value was nevertheless high, indicating excellent ‘rule out’ value in this setting.

## Significant outcomes

- In a consecutive sample of medical inpatients aged 60 and over in Northern Tanzania, the DelApp had good diagnostic accuracy for major cognitive impairment but did not differentiate dementia and delirium
- The DelApp could be administered with individuals too unwell to complete other previously validated cognitive screening tools, allowing identification of those with delirium or reduced arousal and most at risk of negative outcomes

## Limitations

- The high prevalence of uncorrected visual impairment was a major confounder and may limit clinical utility. Adaptations to account for this issue, prevalent in low-middle-income country settings, should be considered.
- This study was completed in a tertiary referral hospital and may not, therefore, be representative of medical inpatient settings across sub-Saharan Africa.

## Introduction

Delirium is an acute onset syndrome of brain dysfunction presenting with deficits in attention, arousal and global cognition (Cerejeira *et al*., 2010). The syndrome is highly prevalent in older hospitalised adults but is greatly underdiagnosed (Inouye *et al*., 2014). Well-evidenced adverse outcomes include cognitive decline (Siddiqi *et al*., 2006; Saczynski *et al*., 2012; Davis *et al*., 2012; Inouye *et al*., 2014), disability (O’Keeffe & Lavan, 1997; Witlox *et al*., 2010) and increased in-hospital and post-discharge mortality rates (Siddiqi *et al*., 2006; Witlox *et al*., 2010). Individuals with pre-existing dementia are at highest risk both of delirium and associated adverse outcomes (Fick *et al*., 2002). Prompt identification can improve outcomes but relies on availability of accurate screening methods.

In sub-Saharan Africa (SSA), demographic transition has resulted in a rapidly growing population of older adults. Recent studies estimate prevalence of dementia and non-communicable disease to be similar to that reported in high-income countries (HICs) (Longdon *et al*., 2013; Guerchet *et al*., 2013; Akinyemi *et al*., 2014) suggesting a population similar to risk of delirium. The current evidence base of delirium in SSA is very limited but suggests a potential high rate of misdiagnosis as psychiatric disorder in older studies (Paddick *et al*., 2015b). In older adults, the limited current data estimate hospital prevalence of delirium at 9.1–19.7% on clinical criteria (Uwakwe, 2000). In contrast, a large case-note-based study reported prevalence of between 0 and 2.6% in older adults admitted to three large centres in Africa, suggesting a large diagnostic gap (Akinyemi *et al*., 2014).

The current lack of validated screening methods for delirium is likely to be a major factor in this diagnosis gap. Cognitive assessment tools developed in HICs frequently perform poorly in SSA due to high levels of illiteracy amongst older adults, especially in rural areas. Another difficulty is that clinicians with specialist skills in delirium assessment including geriatricians, psychiatrists and neurologists are scarce due to a large human resource gap (Bower & Zenebe, 2005; Saxena *et al*., 2007; Dotchin *et al*., 2013). Objective screening methods for the cognitive impairments typical of delirium, which can be used accurately by non-specialists and are not literacy-dependent, are therefore needed. Technological solutions to SSA healthcare resource gaps and smartphone-based apps for screening a range of health conditions are increasingly researched and appear promising (Bright & Pallawela, 2016; Priye *et al.*, 2017, Yeates *et al.*, 2016).

The DelApp is a smartphone-based test of arousal and attention developed in a high-income setting (Edinburgh, Scotland) as an aid to diagnosis and identification of delirium (Tieges *et al*., 2015) and takes less than 5 min to complete. No literacy-dependent items are included, and subjects are simply asked to count visually presented stimuli up to a maximum of 13 items. Published case–control data report both excellent sensitivity and specificity for delirium and the ability to differentiate delirium and dementia (Tieges *et al*., 2014).

### Aims of the study

We aimed to conduct an exploratory study of the utility of DelApp in identification of delirium by non-specialist health workers in an unselected consecutive sample of older medical inpatients in Tanzania. To further evaluate potential utility, we also aimed to compare performance with that of a locally validated brief paper and pencil cognitive test (Identification and Interventions for Elderly Africans [IDEA] 6 item screen) (Paddick *et al*., 2015a) and with the Confusion Assessment Method (CAM) (Inouye *et al*., 1990), a well-validated screening and diagnostic tool for delirium requiring a degree of training and specialist knowledge.

## Materials and methods

### Setting

Kilimanjaro Christian Medical Centre (KCMC) is an 800 bedded tertiary referral hospital in Northern Tanzania, which serves a predominantly rural population of over eight million people across an area of more than 170,000 km^2^. Almost 23,000 admissions were recorded in 2014. KCMC is one of only four tertiary referral centres in Tanzania, and one in six admissions originate from outside the catchment area. The hospital is funded through a partnership between central government and a charitable foundation, with treatments funded through both national health insurance and user payments. This study took place in the 107-bed internal medicine department comprising two inpatient medical wards, a high dependency unit and an eight bedded private ward. A total of 4590 admissions to the department were recorded in 2014 and of these, the proportion of adults aged 60 and over was estimated at 16.1% (Akinyemi *et al*., 2014). In-hospital mortality for those aged 60 and over was estimated at 25.1% in 2012, with a median length of stay of 5 days amongst survivors reflecting the severity of illness in this patient group (Akinyemi *et al*., 2014).

### Ethical approval

Ethical approval was granted locally by the Kilimanjaro Christian Medical College Research and Ethics Committee and by the National Institute of Medical Research of Tanzania in Dar-es-Salaam. Patients were given written and verbal information about the study and its aims before gaining their informed consent. Where patients were unable to write, a thumbprint was used. If patients were admitted unconscious or lacking the capacity to consent, a close relative was able to assent on the patient’s behalf.

### Study design

This study was nested within a 6-month cross-sectional study of prevalence, risk factors and screening methods for delirium in older medical inpatients utilising cross-sectional sampling. Full details of this study have already been published (Lewis *et al*., 2016) but relevant details are repeated here. All individuals aged 60 and over admitted to the medical department during the study period were approached for inclusion. Assessments took place wherever possible from the morning after admission, after initial assessment by the ward medical team. Demographic data collected included self-reported (or informant-reported) literacy, educational and occupational background and sensory impairment.

### DelApp

The DelApp is a clinician-administered smartphone task. It includes a brief ‘behavioural assessment’ intended to measure level of arousal followed by a counting task designed to assess focused and sustained attention. The behavioural test includes ability to keep the eyes open (spontaneously, or on command) for 10 s, follow a simple command (state one’s own name) and visually track the movement of an object (in this case, the smartphone) for ≥5 s. Failure in the behavioural test ends the assessment, and the rater is asked to specify the reason for failure from a predetermined list including lack of cooperation and abnormal level of arousal (drowsiness or restlessness).

A simple visual acuity check is administered prior to the counting task where participants are asked to indicate when a visually presented star changes to a circle. Inability to perceive and communicate this change also ends the test. The remainder of the test consists of six counting trials of up to 13 visually presented star shapes. Participants are asked to count the total number of stars presented in each trial. Maximum trial duration is 30 s. Tasks sequentially increase in difficulty, with distractor shapes (triangles) presented in later trials. Two consecutive failures end the test. Total score is a combination of the arousal score (max score 4) and counting task (attention) score (max score 6). This allows grading of DelApp scores in individuals unable to perform the more challenging sustained attention task. Date and time of test completion are recorded automatically.

The DelApp was administered by two fourth-year medical students (EG and NM, trained by S-MP). The DelApp was administered on a new and encrypted sim-free Android handset purchased for use exclusively for that purpose and downloaded directly to a secure database on completion. No patient-identifiable details were recorded on the handset. Raters aimed to assess all individuals who consented to participate in the main study, on the same day as CAM assessment and were blinded to all other neurocognitive assessments. Blinding was maintained by raters not entering the clinical area during clinical delirium assessments and having no access to clinical notes or study documentation other than a list of consented and eligible participants for assessment provided by the lead researcher on a daily basis.

### Bedside clinical assessment for delirium

All patients were clinically assessed by a research doctor with an interest in geriatrics or psychiatry (S-MP, AD, EGL or LT). Prior to assessment, physical observations and level of arousal were recorded. Where assessment was considered inappropriate due to degree of physical illness or level of consciousness, this was recorded by the assessing doctor and assessment attempted the following day.

Full assessment for delirium included a neurological examination, bedside cognitive assessment and mental state examination. A detailed free-text description of this assessment was recorded. Where possible, bedside cognitive assessment included attention (days of the week backwards), registration, recall and orientation as well as an assessment of long-term memory, receptive and expressive language, praxis, executive function and new learning.

The CAM was scored alongside clinical delirium assessment and used as a guide for the assessing medical team. The CAM has excellent sensitivity and specificity for DSM-IV delirium (Inouye *et al*., 1990) and is frequently used for both diagnosis and screening. The CAM includes assessment of acute onset, fluctuation, inattention, disorganised thinking and altered level of arousal (Inouye *et al*., 1990). Where full CAM assessment was not possible (usually because reduced arousal prevented assessment for attentional deficit or disorganised thought), participants were classified as ‘CAM unable’, and assessment by observation, limited neurological examination and informant history was attempted. All CAM-positive patients and a selection of CAM-negative patients selected through drawing lots were reviewed by a neurologist the same day for a second blinded assessment for delirium.

To assist in identification of prior dementia, an informant history for pre-existing cognitive and functional performance was obtained for all participants. Where low mood was noted on the Mental State Examination, a geriatric depression scale (15-item GDS) was completed to assist in identification of major depression.

Delirium assessment took place blinded to the outcome of the DelApp and IDEA screen, as these data were filed separately and not accessible to the clinical assessment team.

### Consensus diagnoses of delirium and dementia

All clinical data with the exception of DelApp, CAM scoring and the IDEA screen were subsequently reviewed by a consultant old age psychiatrist (EML), a nurse specialist in old age psychiatry (GC) and a research doctor in psychiatry (S-MP). Diagnoses of delirium against DSM-5 criteria were made by consensus. Blinding to CAM and IDEA screen results was maintained to allow formal validation of these tools against DSM-5 criteria for the main study, but also allowed comparison of DelApp with established alternative measures in this study. Consensus diagnoses of dementia were made where there was clear evidence of a dementia syndrome taking into account both clinical assessment and collateral/informant history. Where diagnosis of dementia was likely, but diagnostic criteria were not met, a follow-up clinical interview was offered to participants and informants to clarify diagnosis. Where necessary due to geographical constraints, this interview took place by telephone. Formal dementia subtype diagnoses were limited due to lack of neuroimaging facilities locally.

### Assessment for confounders

Visual acuity was formally assessed through use of a Landholt C broken ring logMAR three-metre chart designed for use in illiterate populations. In individuals unable to see the top line of the chart (6/60 or 1.0 logMAR), an assessment of ability to count fingers and perceive light was conducted. Hearing impairment was graded subjectively as mild, moderate or severe based on performance during clinical assessment. Pain was assessed on a visual analogue scale of 0–10 with 10 rated as most severe. Where necessary, a non-literate or non-verbal assessment of pain was used, and equivalent scores were recorded.

A National Early Warning Scale (NEWS) score was retrospectively calculated using the physical observations taken on the day of assessment. The NEWS is a chart-based assessment of arousal level, respiration rate, oxygenation using pulse oximetry, blood pressure, body temperature and heart rate, which stratifies hospital patients into low-, intermediate- and high-risk clinical groups (Hawkes, 2012). A score of seven or above, defined as ‘high risk’ on the NEWS, was classified as serious physiological illness. Level of arousal was recorded using the Alert-Voice-Pain-Unresponsive (AVPU) scale (American College of Surgeons, 1989) designed for use by non-specialists in routine practice and included within the NEWS score.

### Additional paper and pencil delirium screening

An IDEA six-item cognitive screen was attempted in all consented individuals. This is a low-literacy paper and pencil test developed for dementia screening in SSA, previously validated in hospital and community settings in Tanzania and Nigeria (Paddick *et al*., 2015a; Gray *et al*., 2016; Masika *et al*., 2018). No specific assessment of attention is included. The IDEA was completed by a study nurse or clinical officer after obtaining informed consent, immediately filed securely and outcome was not shared with doctors completing assessments for delirium.

Where the IDEA was attempted but abandoned because of confusion or inability to understand the task, total scores were recorded as zero as the individual was assumed to have severely impaired cognition preventing completion. Where screening was not possible due to physical illness or lowered conscious level, outcome was recorded as ‘unable to complete’.

### Statistical analysis

Data were analysed using PASW version 20 for Windows. All data were non-normally distributed, and therefore, data were presented by median and interquartile range, and non-parametric tests (Mann–Whitney U, Kruskal–Wallis) were used throughout. Chi-square and Fisher’s exact test were used for proportional data. Diagnostic accuracy was analysed using the area under the receiver operative curve statistic (AUROC) as an overall assessment of screening performance alongside sensitivity specificity, positive predictive value, negative predictive value and likelihood ratio. Spearman’s rho was used for non-parametric correlations. A multivariable logistic regression model was constructed using a positive DelApp score as the dependent variable. Predictor variables were first forced into the model using an enter method, and a backwards likelihood ratio model was subsequently constructed to determine the most significant predictors of DelApp score.

Relationships between age, gender delirium diagnosis, educational level, arousal and physical illness were explored in univariable regression analysis prior to construction of the multivariable model. A number of variables were subsequently recategorised as dichotomous for further analysis including education (>/1 year vs no education), AVPU score (alert (A) vs not alert (V/P/U)) and visual acuity (>/6/60 vs. <6/60). Age was recategorised into three bands. Multiple imputation was used for missing visual acuity data using age and self-reported visual impairment and use of visual aids as predictors in a linear regression model based on the outcomes of simple correlations. Data were assumed not to be missing at random, as inability to complete the test correlated with both severe physiological illness and delirium.

The significance level was set at 5% and two-tailed tests were used throughout.

## Results

The study took place between the 23rd of June and 10th of July 2015. During the study period, there were 94 individuals aged 60 and over admitted to the medical department. Seventy-eight were eligible for DelApp screening following successful recruitment to the main study.

Seventy individuals were assessed on admission using the DelApp. DelApp screening took place on the same day as clinical delirium assessment in 66/70 (94%) cases and between 1 and 3 days later in 4/70 (6%) of cases. Eight eligible individuals were not assessed due to resource constraints (student raters were unavailable due to university examinations or other compulsory activities). Analysis was restricted to DelApp assessments taking place on the same day as clinical delirium assessment due to the possibility of a differing clinical picture being present at different time periods.

Of those 66 individuals included, 36 (54.5%) were male. Median age was 75 (IQR 66–82). No significant differences in gender distribution or median age were present between those assessed and all admissions during the study period (chi-square 1.831, sig 0.176, Fisher’s exact test 0.259, MWU 1.88) or between those who were and were not assessed amongst those eligible (Chi 3.545, sig 0.06, Fisher’s exact test sig. 0.114, MWU 0.446).

Demographic data and baseline assessment outcomes are presented in Table 1. The majority of participants (40/66; 66%) were agricultural workers and had primary school education or lower (47/66; 71.2%). Educational background varied widely with 12/66 (18.2%) reporting 1 year or less of schooling, and 8 (12.1%) university-level education.

**Table 1.** Demographic data

Characteristics of the cohort	
Gender % female	30 (45.4%)
Age (Md, IQR)	75 (66–82)
Living alone, (*n*, %)	6 (9.1%)
Marital status	32 (48.5%) married
	31 (47%) widowed
	1 (1.5%) separated
	2 (3%) never married
Occupation rural farmer (*n*, %)	40 (60.6%)
Retired vs still working (*n*, %) (*M* = 3)	12/63 (19.1%)
Highest education level	12 (18.2%) No formal education
	35 (53.1%) primary school level
	11 (16.7%) secondary school level
	8 (12.1%) higher/post-secondary education
Literacy (*n*, %) (*M* = 9)	46 (69.7%)
Self-reported visual impairment (*M* = 2)	20 (30.3%) none
	41 (62.1%) visual impairment
	3 (4.5%) blindness
Pain score at assessment (0–10) (*M* = 1)	4 (2–6)
NEWS score at assessment (Md, IQR)	4 (2.75–6.25)
AVPU (*M* = 1)	47 (71.2%)
	11 (16.7%)
	3 (4.5%)
	4 (6.1%)
HIV status	15 (22.7%) Positive
	3 (4.5%) Negative
	48 (72.7%) Unknown

Prevalence of delirium by DSM-5 consensus criteria was 15/66 (22.7%), dementia 16/66 (24.7%) and delirium superimposed on dementia 7/66 (10.6%). Total prevalence of major cognitive impairment was 24/66 cases (36.4%).

### Screening tool performance

Between-group comparisons of screening tool performance and demographic data are presented in Table 2. Diagnostic accuracy of the DelApp for identification of delirium and major cognitive impairment (dementia and delirium) is summarised in Table 3 and compared to performance of alternative measures (CAM and IDEA).

**Table 2.** Between-group comparisons

	No delirium or dementia (*n* = 42)	Delirium (DSM-5) (*n* = 15)	Dementia without delirium (*n* = 9)	Significance and pairwise comparisons
Age (Md)	73 (65–78)	80 (67–85	80 (76–87.5)	*p* 0.029 (KW)
Gender (F %)	21 (50%)	6 (40%)	3 (33%)	*p* 0.59 (Chi 1.06)
Education (% some)	34 (81%)	13 (87%)	7 (78%)	*p* 0.84 (ns) (Chi 0.357)
AVPU scale (% alert)	38 (90%)	4 (27%)	5 (62.5%), 1 missing	*p* < 0.001 (Chi 19.39)
NEWS SCORE (Md, IQR)	3 (2–6)	5 (4–7)	4 (2–5.5)	*p* 0.019 (KW)
DelApp total score/10 (Md, IQR)	7 (4–10)	4 (2–4)	3 (IQR 2–4)	*p* < 0.001 (KW)
IDEA screen total/15 (Md, IQR)	9 (5–12.5), 1 missing	0 (0–4)	0 (0–5.5)	*p* < 0.001 (KW)
Verbal fluency (animals)/min (Md, IQR)	9 (5.5–11.5), 5 missing	1 (0–4), 3 missing	0 (0–5.5)	*p* < 0.001 (KW)
Stick design test	3 (1–3)	0 (0–0)	0 (0–2)	*p* < 0.001 (KW)
Word learning/30 (Md, IQR)	10 (5–13)	0 (0–5)	0 (0–6.5)	*p* < 0.001 (KW)
Word recall/5	2 (0–3)	0 (0–0)	0 (0–0.5)	*p* < 0.001 (KW)
Is there a sudden change? % yes	8 (21.6%), 5 missing	12 (87%), 1 missing	4 (50%), 1 missing	*p* 0.01 (Chi 17.785)
CAM-positive?	2/42+	10/15+	1/9 CAM+	
	3/42 CAM unable	4/15 CAM unable	3/9 CAM unable	
	37 CAM−	1/15 CAM−	4/9 CAM−	

AVPU, awake, voice, pain, unresponsive scale; CAM, Confusion Assessment Method; F, female; IQR, interquartile range; KW, Kruskal–Wallis test; ChiSq, Chi-squared; Md, median; NEWS, New Early Warning Scale score; NS, non-significant.

**Table 3.** Diagnostic accuracy of tools for measuring delirium and cognitive impairment

	AUROC	Prev.	Sen.	Spec.	PPV	NPV	LR+	LR−
*DSM-5 delirium*								
CAM+ (CAM unable excl)	0.921 (0.814–1)	11/56 (19.6%)	0.909	0.933	0.769	0.977	13.636	0.097
DelApp four and below	0.761 (0.647–0.875)	15/66 (22.7%)	0.867	0.608	0.394	0.939	2.21	0.219
IDEA seven/below	0.799 (0.677–0.921)	10/56 (17.9%)	0.9	0.565	0.310	0.963	2.07	0.177
IDEA five/below		10/56 (17.9%)	0.9	0.652	0.36	0.968	2.588	0.153
*Cognitive impairment (DSM-5 delirium or dementia)*								
CAM+	0.798 (0.651–0.945)	17/56 (30.4%)	0.647	0.949	0.846	0.860	12.618	0.372
DelApp four and below	0.834 (0.735–0.933)	24/66 (36.4%)	0.813	0.789	0.636	0.909	3.06	0.175
IDEA seven/below	0.843 (0.734–0.953)	17/56 (30.4%)	0.824	0.615	0.483	0.889	2.141	0.287
IDEA five/below		17/56 (30.4%)	0.824	0.718	0.56	0.903	2.92	0.246
*DSM-IV dementia*								
CAM+	0.695 (0.504–0.886)	11/56 (19.6%)	0.545	0.844	0.462	0.884	3.506	0.538
DelApp four and below	0.784 (0.667–0.902)	16/66 (24.2%)	0.875	0.620	0.424	0.939	2.303	0.202
IDEA seven/below	0.814 (0.680–0.949)	12/56 (0.214)	0.833	0.568	0.345	0.926	1.93	0.293
IDEA five/below		12/56 (0.214)	0.833	0.659	0.4	0.935	2.44	0.253

### Performance of DelApp

The DelApp identified delirium with sensitivity of 0.87 and specificity of 0.62 (AUROC 0.77) at the optimum cut-off in this study of 4/10 (ability to complete the behavioural assessment) (*n* = 66). In identification of major cognitive impairment (including both delirium and dementia by DSM-5 criteria), the DelApp was more accurate (sensitivity 0.88, specificity 0.73, AUROC 0.834).

In comparison, 85% (56/66) of participants completed CAM assessment but 10 (15.3%) were ‘CAM unable’. Of those assessed, diagnostic accuracy vs DSM-5 was excellent (AUROC 0.93).

The IDEA was completed by 56/66 (85%) of participants, including two judged unable to continue due to severe cognitive impairment and scored 0/15. Diagnostic accuracy was good for major cognitive impairment (AUROC 0.843 (95% CI 0.73–0.95)) but fair for delirium (AUROC 0.799 (95% CI 0.68–0.92)). Notably, the optimal cut-off in this cohort was substantially lower than in previous hospital and community studies (5/15 vs. 7/15).

Evidence of concurrent validity of the DelApp in measurement of cognitive impairment included significant positive correlations at the 0.01 level with the IDEA six-item cognitive screen [Γ 0.770, sig <0.001] and bedside tests of registration [Γ 0.561, sig <0.001], recall [Γ 0.653] orientation [Γ 0.657, sig <0.001] and sustained attention (days of the week backwards) [Γ 0.672, sig <0.001]. The DelApp was also highly correlated with a positive CAM score amongst those participants able to complete it [ 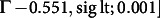 .

**Figure 1.**
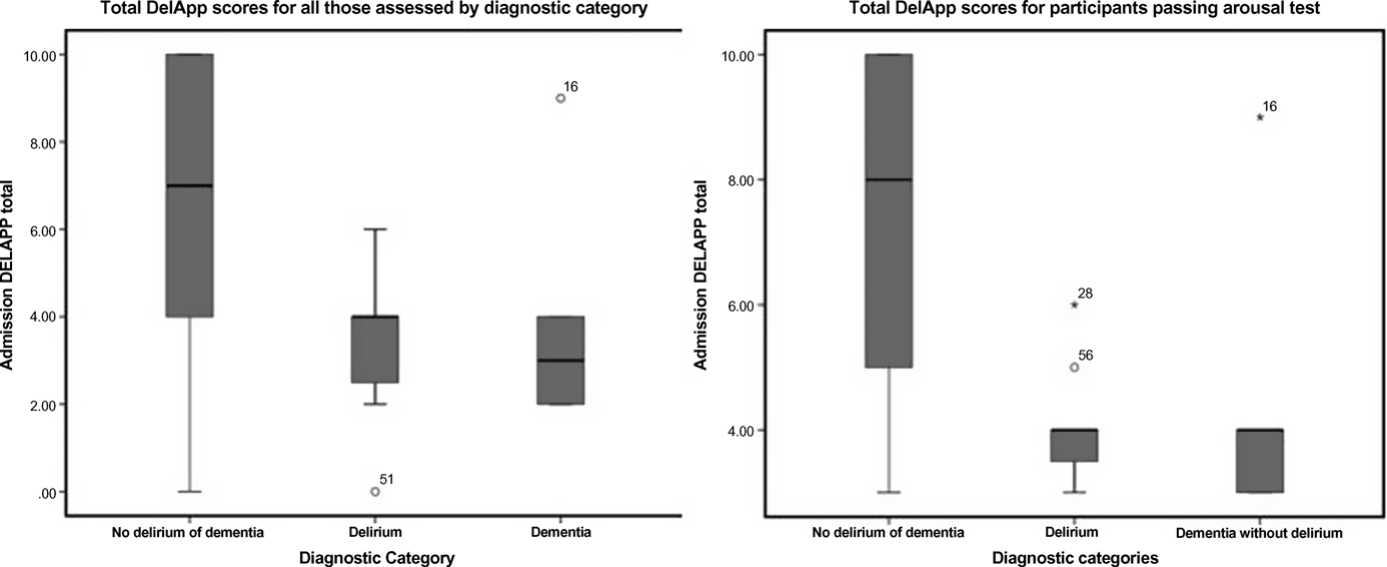
Boxplot of total Delapp scores for no delirium or dementia, delirium and dementia without delirium groups.

Median score on the DelApp was 7/10 in those without delirium or dementia, four (IQR 2–6) in delirium (including individuals with underlying dementia) and three (IQR 2–4) in individuals with dementia but no delirium (see Fig. 1). Pairwise comparisons were significant between those with no major cognitive impairment and those with delirium or dementia, but not between the delirium and dementia groups.

The DelApp successfully graded all participants for arousal and inattention, whereas other screening tests could not be completed due to lowered arousal. All ten ‘CAM unable’ participants were assessed using the DelApp. Of these, only 3/10 successfully completed the behavioural/arousal test with others failing through inability to cooperate or altered arousal, but all were graded. Likewise, of the 10 individuals not assessable by six-item screen, all were assessed using the DelApp. Similarly, only 3/10 passed the behavioural/arousal test (four due to altered arousal and three due to inability to cooperate).

Reasons for failing the arousal test included drowsiness (four cases), restlessness and agitation (one case) or inability to cooperate (six cases). When analysis was restricted to those passing the DelApp arousal test, median score increased to eight (IQR 5–10) in those without delirium or dementia, and diagnostic accuracy improved (AUROC 0.81 (95% CI 0.696–0.926)). A significant proportion of those passing the arousal test (15/56 (22.7%)) failed the visual acuity test and did not complete the counting (sustained attention) task. Half of these had delirium and in half, visual acuity was not assessable. Of those with assessable visual acuity, only two had acuity of >/6/60.

### Association with confounders

There was a significant prevalence of uncorrected sensory impairment. Of those able to be assessed, half (22/44) had visual acuity of less than 1.0 (6/60) in the best eye. Three patients (6.8%) were unable to count fingers in the best eye. In a third of patients (22/66), vision could not be objectively assessed due to abnormal cognition, arousal or degree of physical illness preventing completion of the visual acuity test. The majority of participants self-reported significant visual impairment (41/64 (64.1%)) and 3/64 (4.5%) reported blindness. One-third (21/66) owned reading glasses, but none used glasses for distance vision.

DelApp total score showed a significant negative correlation with age [Γ 0.389, sig 0.001], NEWS score [Γ −0.336, sig 0.006] and lowered arousal on the AVPU scale [Γ −0.601, sig 0.00] but did not correlate with pain measured on the visual analogue scale. DelApp score was also significantly positively correlated with visual acuity of 6/60 or better [Γ 0.445, sig 0.004], but not with self-reported visual impairment. There was no significant correlation between DelApp score and highest educational level or history of any formal education, but a modest correlation at the 0.05 level with literacy [Γ 0.314, sig 0.017].

In contrast, delirium diagnosis positively correlated with NEWS score [Γ 0.341, sig 0.05] and reduced arousal [Γ 0.517, sig 0.00] measured on the AVPU scale but not with objective or subjective visual impairment, educational level, literacy or age.

**Figure 2.**
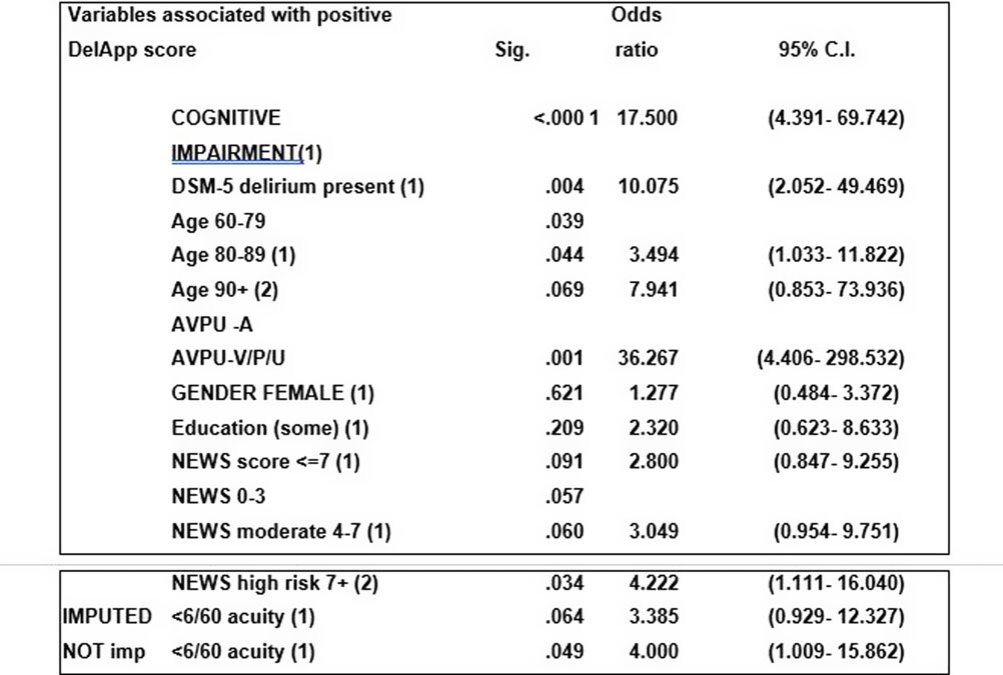
Univariable logistic regression model with dichotomised positive DelApp score as the dependent variable.

**Figure 3.**
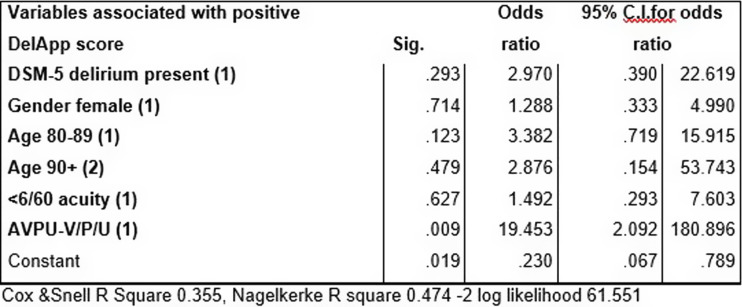
Multivariable logistic regression model with positive DelApp score dichotomised as the dependent variable.

When factors likely to be associated with DelApp performance were analysed in a univariable logistic regression model with positive DelApp screen as the dependent variable (see Fig. 2), significant associations with increased age, delirium diagnosis, major cognitive impairment and reduced arousal were present. On multivariable analysis (where all covariates were forced into the model), only reduced arousal remained significant (see Fig. 3). Likewise, where a backwards likelihood model was used, arousal level alone was the best predictor of positive DelApp score after four iterations (OR 36.27, 95% CI 4.40–298.53, sig 0.001).

## Discussion

In this study, performance of the DelApp in identification of major cognitive impairment was similar to that of a previously validated paper and pencil cognitive screen (the IDEA six-item screen). In identification of delirium, performance was inferior to the CAM in those able to complete it, but the CAM was not able to be used in a third of participants and a number of diagnoses of delirium were missed.

In a previously published case–control study, the DelApp (Tieges *et al*., 2015) was able to discriminate between delirium and dementia with individuals with mild-to-moderate dementia noted to be able to complete the sustained attention (counting) task in contrast to those with delirium who could not. In this cohort, the DelApp was unable to distinguish between delirium and dementia and median scores were similar between groups. Sensitivity was high for delirium but specificity was low due to false positives with dementia or reduced consciousness without delirium. Although impaired attention is a fundamental part of the delirium syndrome, other tests of attention frequently show poor discrimination between delirium and dementia (Adamis *et al*., 2016) and similarly high sensitivity but poor specificity. Abnormal arousal is specific but not sensitive to delirium.

Median DelApp scores were also very low and markedly lower than those reported in a previously published case–control study (Tieges *et al*., 2015). Most individuals with delirium or dementia in our study were not able to correctly complete any of the counting trials. A significant proportion of individuals were not able to correctly complete the visual acuity pre-test, which required identification of shapes presented on the smartphone screen. It appears likely that this was due in part to reduced visual acuity in this cohort, but may have been due in part to a degree of inattention and cognitive impairment.

The strongest predictor of performance on the DelApp was altered (reduced) arousal, and the prevalence of reduced arousal was high in our cohort. Not all of these individuals met DSM-5 delirium criteria. A high rate of impairment of consciousness (59%) separate from delirium (20%) was present in a large cohort of neurological patients in another large hospital in Tanzania and associated with 27% in-hospital mortality (Winkler *et al*., 2011). Reduced arousal at hospital admission has also been associated with increased mortality and morbidity independent of presence of delirium (Han *et al*., 2014). Altered arousal forms part of the major differences between DSM-IV and DSM-5 delirium criteria, whereas DSM-IV specified altered arousal in the diagnostic criteria, DSM-5 refers instead to disturbances in attention. Strict application of the criteria might therefore lead to individuals with significantly reduced arousal being misdiagnosed, as attention cannot be assessed. Guidelines from the European Delirium Association (European Delirium Association, 2014) warn against this and advise that both arousal and attention are key elements of consciousness and that reduced arousal should be regarded as ‘severe inattention’ given that below a certain level of arousal, attentional impairment cannot be assessed.

A major advantage of the DelApp was that it could be attempted in all participants and the inclusion of the arousal task allowed grading of those with high levels of inattention. In contrast, the CAM and six-item screen were judged unassessable in a significant proportion of individuals, primarily due to lowered arousal or degree of physical illness. Therefore, potential advantages were that the absence of a floor effect meant that participants unable to complete the more difficult cognitive screening tools could be assessed and the extent of attentional deficits graded. In this setting with a high prevalence of acute physiological illness and impairment of consciousness, the DelApp would be useful in identifying those with cognitive impairment needing further assessment for delirium and also in those with significantly reduced arousal. Since arousal is an independent predictor of morbidity and mortality even in the absence of delirium, use of the DelApp could be useful in identifying those at risk, in measuring the extent of attentional deficits and possibly delirium severity.

A number of limitations are acknowledged. KCMC is a tertiary referral hospital and therefore those admitted would be expected to be more seriously unwell than in other hospital settings. Educational level was markedly higher than that recorded in previous validation studies of the IDEA six-item screen in the same geographical region, indicating possible differences in socio-economic status. Our cohort might therefore not be typical of other settings in Tanzania. This was a small study, and individual subgroups as outlined in Table 2 include small numbers, making firm conclusions challenging.

All cognitive tests were conducted in a very busy ward environment, which could at times be noisy, and this could have impacted performance on the DelApp and also other cognitive tests. Nevertheless, no private or quiet environment for testing was available, and this, therefore, represented the ‘real-life’ conditions in which the DelApp would normally be used.

Although we have data on visual acuity for those able to be assessed, a substantial proportion of participants were unable to cooperate with visual acuity testing. We also have no data on the prevalence of uncorrected cataract, which may have interfered with ability to perceive the visual stimuli on the DelApp. The degree to which visual impairment may have impacted performance remains unclear. It should be noted that the original DelApp studies excluded those with visual impairment. We felt however that exclusion of people with visual impairment would be impractical in routine clinical practice in sub-Saharan Africa and to do so in this study would limit generalisability. Visual impairment might therefore be considered a potential limitation of this screening method in settings with high level of untreated cataract.

Completion of DSM-5 delirium diagnoses relied upon clinical assessment data including bedside cognitive assessment. Where participants had markedly reduced levels of arousal and could not be assessed, clinical assessment was limited. It is possible that a number of those with reduced arousal with delirium may have been missed, as insufficient information was present.

Not all those eligible for the study were assessed with the DelApp, but we have no reason to believe that those who were and were not assessed differed significantly.

This was not a study of dementia or depression, and therefore milder cases may have been missed, particularly in those with delirium at the time of assessment. Nevertheless, we were able to obtain an informant history in all participants, and the vast majority of participants lived with family members. As a result, cognitive impairments were likely to have been observed and commented on by family members in the history.

Arousal was measured by AVPU and GCS, which focus on lowered arousal, whereas delirium criteria include increased arousal or agitation. Inclusion of a brief arousal scale such as the Richmond arousal assessment (Morandi *et al*., 2016) would have allowed concurrent validity with the DelApp to be measured, and the relationship between altered and not simply lowered arousal and DelApp score to be explored.

Despite the identified limitations, strengths of the study were the broad inclusion criteria potentially representative of other similar medical inpatient settings in sub-Saharan Africa and the extensive clinical and neurological assessment including an informant history to support consensus panel diagnosis. Studies of delirium in older people in sub-Saharan Africa are few. This study suggests that in this setting, the DelApp had good diagnostic accuracy in identification of major cognitive impairment (but did not differentiate delirium and dementia) and could increase detection of those with delirium or lowered arousal in routine clinical care. The identified issues around untreated visual impairment suggest adaptations for SSA should be considered.
